# Mesoporous carbon-containing voltammetric biosensor for determination of tyramine in food products

**DOI:** 10.1007/s00216-016-9612-y

**Published:** 2016-05-21

**Authors:** Jolanta Kochana, Karolina Wapiennik, Paweł Knihnicki, Aleksandra Pollap, Paula Janus, Marcin Oszajca, Piotr Kuśtrowski

**Affiliations:** Faculty of Chemistry, Jagiellonian University, Ingardena 3, Krakow, Poland

**Keywords:** Biosensor, Tyramine, Food, Mesoporous carbon, Tyrosinase, Voltammetry

## Abstract

A voltammetric biosensor based on tyrosinase (TYR) was developed for determination of tyramine. Carbon material (multi-walled carbon nanotubes or mesoporous carbon CMK-3-type), polycationic polymer—i.e., poly(diallyldimethylammonium chloride) (PDDA), and Nafion were incorporated into titania dioxide sol (TiO_2_) to create an immobilization matrix. The features of the formed matrix were studied by scanning electron microscopy (SEM) and cyclic voltammetry (CV). The analytical performance of the developed biosensor was evaluated with respect to linear range, sensitivity, limit of detection, long-term stability, repeatability, and reproducibility. The biosensor exhibited electrocatalytic activity toward tyramine oxidation within a linear range from 6 to 130 μM, high sensitivity of 486 μA mM^−1^ cm^−2^, and limit of detection of 1.5 μM. The apparent Michaelis–Menten constant was calculated to be 66.0 μM indicating a high biological affinity of the developed biosensor for tyramine. Furthermore, its usefulness in determination of tyramine in food product samples was also verified.

**Electronic supplementary material:**

The online version of this article (doi:10.1007/s00216-016-9612-y) contains supplementary material, which is available to authorized users.

**Graphical abstract:**
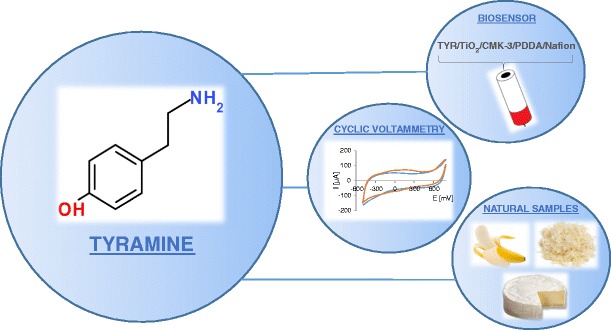
Different food samples were analyzed to determine tyramine using biosensor based on tyrosinase

## Introduction

Tyramine (*p*-hydroxyphenylethylamine), being a decarboxylation product of tyrosine, belongs to the group of biogenic amines [1]. This compound can be found in many food products (e.g., chocolate, wine, beer, cheese, beans, banana peel, ketchup, fish) and can be considered as their quality indicator, because the concentration of tyramine increases in perishable products [2, 3]. Consumption of a large amount of tyramine-containing food has a toxic effect on health producing symptoms such as flush, rash, vomiting, palpitation, tachycardia, and hypertonia [4]. Furthermore, it can lead to a dangerous blood pressure increase, accompanied by an attack of strong migraine headaches [5–7]. For these reasons, the development of sensitive, selective, and inexpensive methods for the determination of tyramine in food products is really an important issue.

There are some known methods for the determination of tyramine in various types of food. Most commonly, chromatographic methods for quantification of tyramine and other biogenic amines were reported, exemplary high-performance liquid chromatography [7–9], hydrophilic interaction liquid chromatography with tandem mass spectrometry (HILIC–MS/MS) [10], and ultra-performance convergence chromatography [11]. Moreover, capillary electrophoresis–tandem mass spectrometry [12] and capillary electrophoresis coupled with electrochemiluminescence [13] were tested. Nevertheless, most of them require complicated sample preparation, and they are expensive and usually time-consuming. Electrochemical (bio)sensors turned out to be a favorable alternative for the quantification of tyramine because of their simplicity, selectivity, and rapidity. Additionally, they do not require time-consuming sample pretreatment and expensive instrumentation. Recently, few types of electrochemical biosensors for the determination of tyramine have been described in the literature, including those using as an enzyme pea seedling amine oxidase [4, 14] or black gram tyramine oxidase [2, 15]. Electrochemical biosensing of tyramine based on tyrosinase was reported only twice. Apetrei at al. developed a biosensor with tyrosinase immobilized in phosphate-doped polypyrrole film [16] and produced the screen-printed electrode with enzyme entrapped in a carboxyl-functionalized single-walled carbon nanotube composite [17].

Besides carbon nanotubes [18], graphene [19], and fullerenes [20], ordered mesoporous carbons (OMC) are often tested as components of novel biosensors. Among OMC, the most frequent CMK-3 and CMK-1 are employed for biosensor matrix preparation. The CMK-n-type replicas have gained the interest of scientists since the end of the 1990s [21, 22]. The good electronic conductivity, highly defined pore size distribution, high specific surface area, large pore volume, and enhanced mechanical stability cause them to be useful in catalysis, adsorption, and electrochemical applications, like a construction of chemical as well as biochemical sensors. For example, the CMK-3-type replica is characterized by the hexagonal arrangement of carbon nanorods with the specific surface area in the range of 900–1500 m^2^ g^−1^, total pore volumes of 1.1–1.7 cm^3^ g^−1^, and pore sizes ranging between 3.3 and 5.0 nm [23]. The widespread use of mesoporous carbon materials is noted in construction of amperometric, potentiometric, and voltammetric chemical sensors dedicated to the determination of metals [24], dopamine [25], glucose [26], hydrogen peroxide [27], NADH [28], explosive materials (TNT) [29], uric acid [30], and others. CMK-3 was also successfully used in preparation of a (GCE/{chitosan/hemoglobin–CMK-3}_6_) multilayer film-modified biosensor for hydrogen peroxide determination [31]. Furthermore, a functionalized CMK-3, synthesized using SBA-15 as the template and sucrose as the carbon source, has been found to be practical in the determination of toxic ractopamine in pork [32]. Direct electroanalysis of double-stranded DNA after accumulation and preconcentration on OMC-modified GCE-film electrode was presented by Wang et al. [33].

In this work, we present a voltammetric tyrosinase-based biosensor for the determination of tyramine. Tyrosinase (TYR) catalyzes oxidation of tyramine to corresponding *o*-quinone, which, being reduced at the electrode surface, generates current corresponding to the tyramine concentration (Scheme S1; see Supplementary Information). For the entrapment of enzyme titania sol–gel (TiO_2_), modified with a carbon material, polycationic polymer poly(diallyldimethylammonium chloride) (PDDA) and Nafion were employed. Two different carbon materials were compared as the matrix components: multi-walled carbon nanotubes (MWCNT) and CMK-3-type mesoporous carbon. Our earlier research led to fabrication of a TYR/TiO_2_/MWCNT/PDDA/Nafion biosensor that exhibited good analytical characteristics, particularly in terms of sensitivity [34]. CMK-3 was taken into account as the biosensor matrix component due to its promising features related to the mesoporous structure. To characterize the properties of selected immobilized composites, scanning electron microscopy (SEM) and cyclic voltammetry (CV) were applied. The resulting biosensor was evaluated in terms of analytical parameters such as linear range, sensitivity, limit of detection, long-term stability as well as repeatability and reproducibility. Functioning of the proposed biosensor was checked in determination of tyramine in food samples (Camembert cheese, sauerkraut, and banana) with satisfactory results.

## Experimental

### Chemicals and materials

Tyramine (99 %), glutathione (99 %), phenylalanine (98 %), titanium(IV) isopropoxide, tyrosinase from mushroom (EC1.14.18.1; 5771 U mg^−1^), vanillic acid, gallic acid, and poly(diallyldimethylammonium chloride) PDDA (average Mw < 100,000, 35 wt% in H_2_O) were purchased from Sigma-Aldrich (USA); tryptophan (98.5–101 %) and catechol were from Merck (Germany) and caffeic acid was from Koch-Light Laboratories; HCl (35 %), ethanol (96 %), 2-propanol, and l-(+)-ascorbic acid were obtained from Avantor Performance Materials Poland S.A. (Poland); HNO_3_ (65 %), NH_3_(aq) (25 %), and acetone were purchased from LACHNER (Czech Republic); 0.3 mm alumina powder was from Buehler Micropolish (USA); Nafion (5 % (*w*/*v*) solution in mixture of low aliphatic alcohol and water) was obtained from Fluka. Furfuryl alcohol (98 %, Aldrich), hydrochloric acid (33 %, Polish Chemical Reagents), and iron(III) nitrate nonahydrate (Polish Chemical Reagents) were used for CMK-3 synthesis without any further purification. Phosphate buffer solutions (PBS) of pH 6.0 and 7.0, in a concentration of 0.1 M, were prepared by mixing appropriate volumes of KH_2_PO_4_ and Na_2_HPO_4_ solutions. Tyrosinase (0.048 mg/10 μL) and tyramine solution were prepared in 0.1 M phosphate buffer solution, pH 7. All chemicals were analytical-grade reagents. Milli-Q water was used throughout.

### Preparation of CMK-3

Two different carbon materials were used in the preparation of biosensors: (i) commercially available multi-walled carbon nanotubes (MWCNT, Aldrich) and (ii) CMK-3-type carbon replica synthesized by the hard templating method using SBA-15 mesoporous silica (the complete synthesis protocol and characterization of SBA-15 are described in [35]). For the synthesis of CMK-3 material, 3.0 g of SBA-15 was introduced into 94.0 g of distilled water containing 6.0 g of furfuryl alcohol (FA). Subsequently, hydrochloric acid was added at the HCl/FA molar ratio of 6:1 and the slurry was heated up to 100 °C. This temperature was kept for the next 6 h under vigorous stirring to ensure complete polycondensation resulting in precipitation of poly(furfuryl alcohol) (PFA) in the pore system of SBA-15. After that time, the obtained brown solid was filtered, washed with distilled water, and dried at room temperature overnight. Then, the PFA/SBA-15 composite was impregnated with an aqueous solution of iron(III) nitrate by the incipient wetness technique. The amount of introduced Fe^3+^ cations was selected to achieve the Fe concentration of 2 wt% in the PFA/SBA-15 composite. After drying at 40 °C, the material was calcined under inert atmosphere (N_2_, 40 mL min^−1^) at 850 °C with a heating rate of 1 °C min^−1^ and an isothermal period of 4 h. Finally, the silica template was removed by treatment with 5 % hydrofluoric acid solution.

### Preparation of biosensor

The first step of biosensor fabrication was cleaning of a graphite electrode surface by polishing with the use of alumina powder suspension and activating by sonication in different media. The titania sol was synthesized by acid hydrolysis and further polycondensation of titanium(IV) isopropoxide. The detailed procedures of these processes were reported in [36]. For the preparation of the matrix composite, the carbon material (MWCNT or CMK-3) was weighted (the mass depended on the number of produced biosensors) and mixed with a tyrosinase solution. Then, the mixture of titania sol and PDDA solution was added to the CMK-3/TYR (or MWCNT/TYR) suspension and shaken on the vortex. Finally, Nafion was gradually added and the mixture was shaken after each portion. The resulting composite was sonicated in an ultrasonic bath for 5 min.

**Scheme 1:**
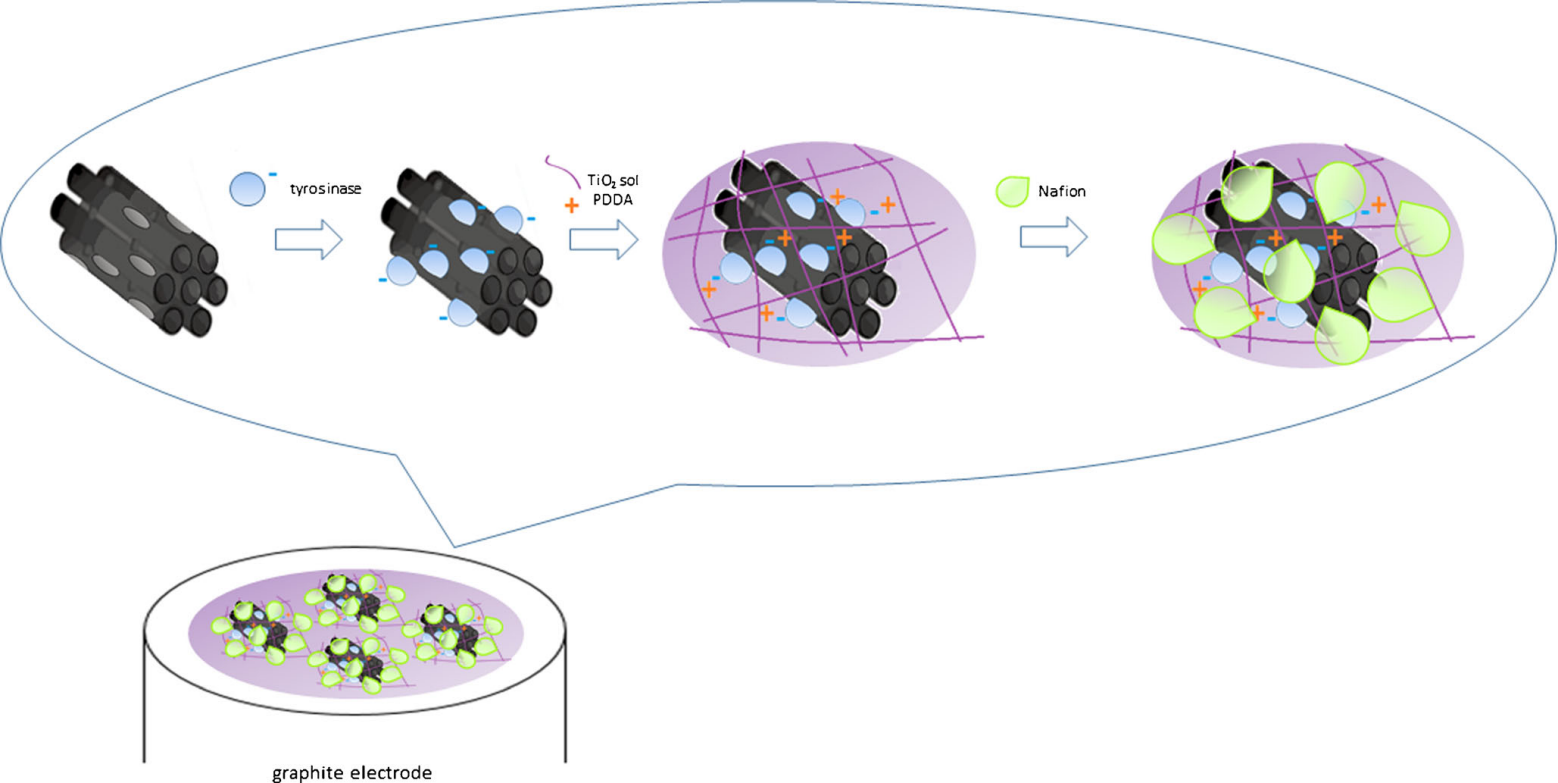
Schematic illustration of TYR/TiO_2_/CMK-3/PDDA/Nafion biosensor preparation

To prepare the biosensor, two 10-μL portions of matrix composite were deposited on the previously prepared surface of graphite electrode and dried after each portion for ca. 10 min. Then, the electrodes were left to dry above a saturated disodium phosphate solution at 4 °C for 20 h. The biosensors were stored at 4 °C in 0.1 M PBS (pH 6). Preparation of the biosensor is schematically presented in Scheme 1.

Twenty microliters of matrix layer deposited on the electrode surface contained (per electrode) 0.041 mg CMK-3, 0.039 mg TYR, 1 μL PDDA, and 5.7 μL Nafion.

### Experimental setup

The measurement technique was cyclic voltammetry conducted with the use of an M161 electrochemical analyzer (mtm-anko, Poland) in a thermostatic cabinet (ST1, Pol-Eko Aparatura, Poland). All measurements were carried out in 0.1 M phosphate buffer solution (PBS, pH 6) as supporting electrolyte. The conventional three-electrode system was employed with the carbon material-modified bioelectrode as the working electrode, the saturated silver/silver chloride reference electrode, and the platinum wire auxiliary electrode (mtm-anko, Poland). The experiments were conducted at a temperature of 25 °C, in a potential range from −0.5 to 0.8 V and at a scan range of 62.5 mV s^−1^.

A vortex mixer (Labnet, USA) and a Sonic 3 Ultrasonic bath (Polsonic, Poland) were used to prepare homogenous matrix composites and extracts of food products (banana, sauerkraut, and Camembert cheese). A homogenizer (WITKO, Poland) and centrifuge (MPW Med. Instruments, Poland) were used for preparation of food samples. The pH of phosphate buffer solutions was measured with the use of a combined glass electrode, ERH-11 (HYDROMED, Poland), and a CP-501 pH-meter (Elmetron, Poland).

The structure of the carbon materials was examined by X-ray powder diffraction (XRD) using a Bruker D2 Phaser instrument with a LYNXEYE detector. The diffraction patterns were collected using Cu Kα radiation (*λ* = 1.54184 Å) in the 2*θ* range of 0.75–70.00° with the 0.02° step. The textural properties of these materials were investigated by means of low-temperature adsorption of nitrogen (−196 °C) in an ASAP 2020 instrument (Micromeritics). SEM pictures were taken with a VEGA3 LM scanning electron microscope (TESCAN ORSAY HOLDING).

### Sample preparation

Three food products were tested in respect of tyramine content: sauerkraut, banana, and Camembert cheese. All of them were prepared for analysis in the same way. To 6.0 g of homogenized sample, 8 mL of phosphate buffer solution (0.1 M, pH 7) was added, mixed in a vortex for 5 min, and sonicated for 10 min. Thereafter, the mixture was centrifuged at +18 °C for 10 min at 10,000 rpm and the supernatant was decanted and centrifuged once again at the same conditions. Finally, the extract was made up with phosphate buffer solution to a volume of 10 mL in a volumetric flask.

### Measurement procedure

Determination of tyramine was conducted in a glass electrochemical cell of volume 20 mL. During electrochemical measurements, cyclic voltammograms were registered and the redox peaks related to enzymatic and non-enzymatic transformations of tyramine were noticed. Both enzymatically generated anodic and cathodic peak currents, as an analytical signal, were investigated. Finally, for analytical purposes, the anodic peak current, reveled at ca. 200 mV, was selected (see section “Evaluation of analytical parameters of biosensor”). The response time of the proposed biosensor was decided to be 3 min; however, an increasing current was recorded for 10 min. The shorter response time was selected due to relatively high values of signal noticed after this period of time and due to the short time of the analysis procedure. The phenomenon of signal growth was noticed in our earlier studies resulting in the development of a biosensor for bisphenol A determination [34]. Because of possible matrix effects, analyses were carried out using the standard addition method.

## Results and discussion

### Structural and textural characterization of MWCNT and CMK-3

**Fig. 1:**
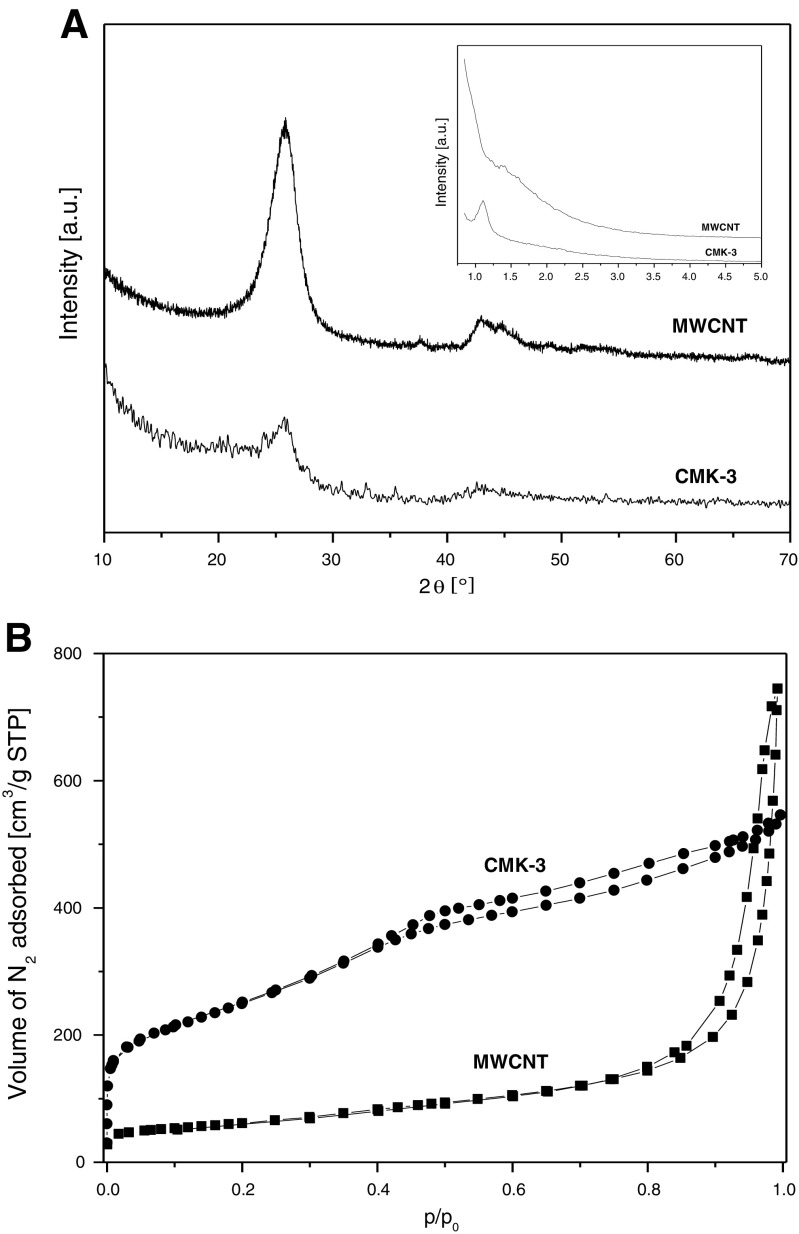
X-ray diffraction patterns (**A**) and N_2_ adsorption–desorption isotherms (**B**) for MWCNT and CMK-3

Both materials (MWCNT and CMK-3), chosen as the carbon matrix, show the presence of diffraction peaks at about 26° and 43°, which can be identified as reflections from the (002) and (100) planes of carbon in the graphite-like structure (Fig. 1A). Obviously, this structure has a significantly higher order of crystallinity for MWCNT. Furthermore, CMK-3 exhibits the diffraction peak at 1.1° (compare inset to Fig. 1A) that can be indexed to (100) representing well-ordered hexagonal pores.

The differences in porosity of both carbon materials are clearly visible in the shape of N_2_ adsorption isotherms presented in Fig. 1B. Nitrogen adsorbs on the MWCNT surface preferentially at high relative pressures suggesting the presence of interparticle cavities, which play a dominant role in the porosity of this material. The BET surface area of MWCNT (*S*_BET_ = 217 m^2^ g^−1^) is therefore rather low. On the other hand, CMK-3 possesses a huge amount of mesopores, as was tentatively suggested by XRD. Their presence is additionally manifested by the typical type IV isotherm with the N_2_ condensation step at *p*/*p*_0_ = 0.3–0.5. Consequently, *S*_BET_ of CMK-3 (893 m^2^ g^−1^) is considerably higher compared to MWCNT.

### Preliminary voltammetric studies. Choice of carbonaceous component of matrix composite

**Fig. 2:**
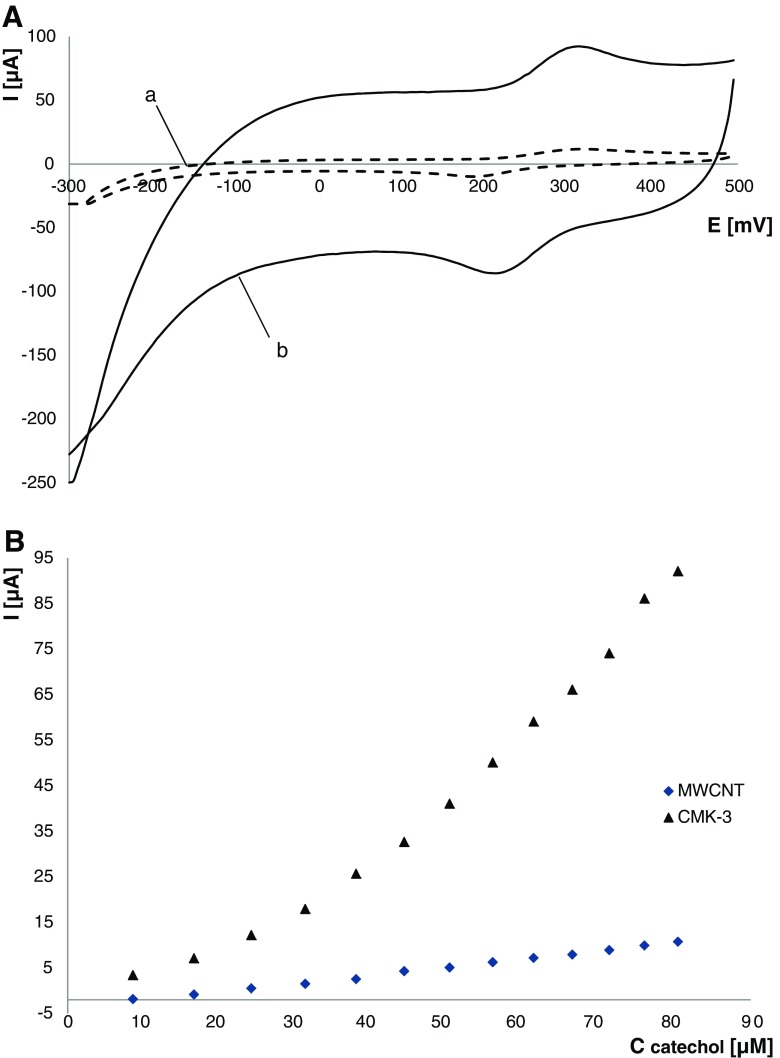
CV curves recorded for TYR/TiO_2_/MWCNT/PDDA/Nafion (**a**) and TYR/TiO_2_/CMK-3/PDDA/Nafion (**b**) biosensors in 45 μM catechol solution (**A**) and corresponding calibration curves (**B**); scan rate, 62.5 mV s^−1^

Preliminary electrochemical experiments were performed to verify whether using CMK-3 as the component of the TYR/TiO_2_/PDDA/Nafion matrix composite improves the analytical properties of the developed biosensor. For that purpose, the bioelectrode with matrix based on CMK-3 was compared with that based on multi-walled carbon nanotubes, MWCNT. The electrochemical behavior of biosensors was examined toward catechol, a model tyrosinase substrate [37]. Figure 2 shows CV curves recorded in 45 μM catechol solution at TYR/TiO_2_/MWCNT/PDDA/Nafion and TYR/TiO_2_/CMK-3/PDDA/Nafion biosensors. The comparison of the obtained CV and corresponding calibration curves clearly indicates that the higher response is noticed for the matrix containing CMK-3. That phenomenon could be explained by the significantly higher surface area of CMK-3, compared to MWCNT (see section “Structural and textural characterization of MWCNT and CMK-3”), and the presence of a huge amount of large-sized mesopores providing homogenous distribution of matrix components. For that reason, the TiO_2_/CMK-3/PDDA/Nafion composite was chosen as the immobilization biosensor matrix for further study.

### SEM characterization of TiO_2_/CMK-3/PDDA/Nafion matrix

**Fig. 3:**
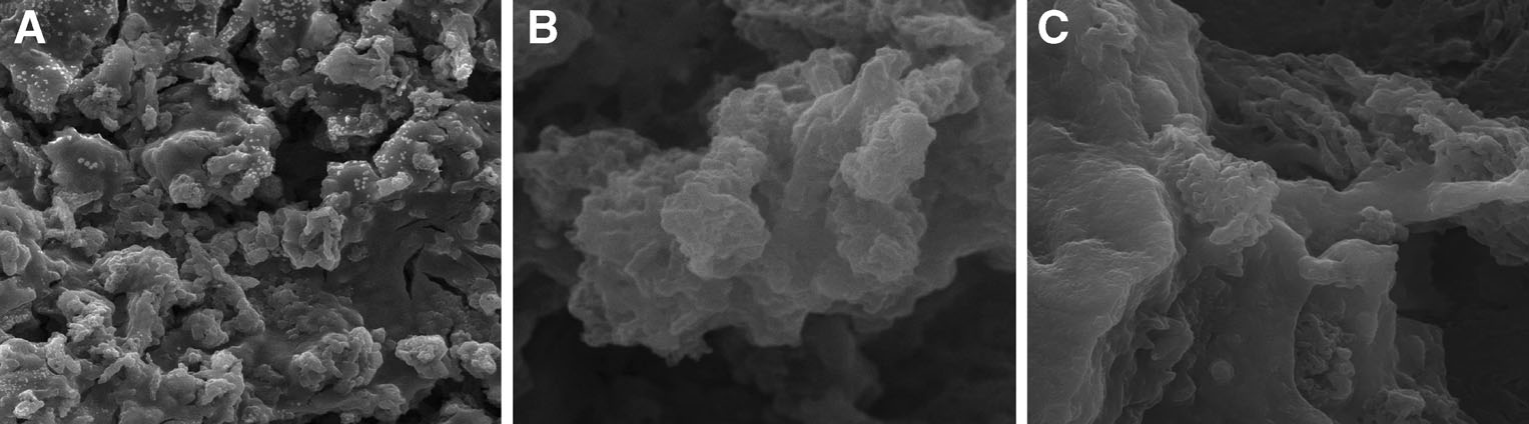
SEM images of TYR/TiO_2_/CMK-3/PDDA (**A**), TYR/TiO_2_/CMK-3/Nafion (**B**), and TYR/TiO_2_/CMK-3/PDDA/Nafion (**C**) composites supported on the graphite electrode surface

The morphology of matrices containing TYR/TiO_2_/CMK-3 modified with PDDA, Nafion, and both of them was characterized using scanning electron microscopy. As can be seen in Fig. 3, the structure of the TYR/TiO_2_/CMK-3 matrix composite enriched with PDDA (Fig. 3A) is flatter than that with addition of Nafion (Fig. 3B). The presence of Nafion affected the tightening edge of the structure. Modification with both components, PDDA and Nafion, resulting in the TYR/TiO_2_/CMK-3/PDDA/Nafion matrix, increases the porosity of the composite enhancing the active surface area for enzyme immobilization. Consequently, the CMK-3/TYR/TiO_2_/PDDA/Nafion composite due to its roughness, porosity, and well-defined structure can be expected to be attractive when the construction of the electrochemical biosensor is considered.

### Electrochemistry of TYR/TiO_2_/CMK-3/PDDA/Nafion biosensor

**Fig. 4:**
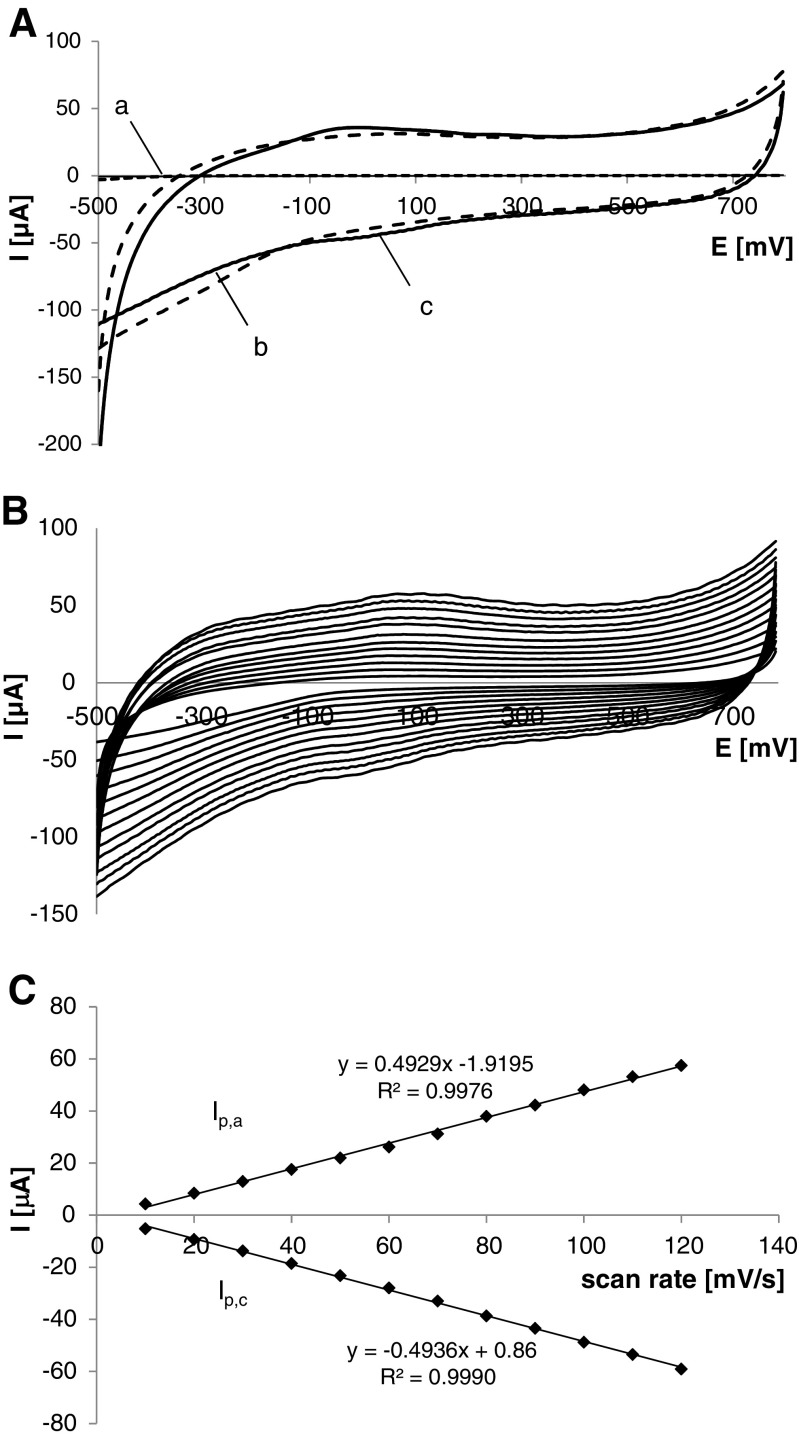
CV curves registered in solution of supporting electrolyte (0.1 M PBS, pH 6) at bare (**a**), TiO_2_/CMK-3/PDDA/Nafion (**b**), and TYR/TiO_2_/CMK-3/PDDA/Nafion (**c**) electrodes (**A**); effect of scan rates (from 0 to 120 mV s^−1^) on cyclic voltammogram (**B**), and dependence of anodic (*I*_p,a_) and cathodic (*I*_p,c_) peak potential versus scan rates (10–120 mV s^−1^) (**C**)

Electrochemical studies on the developed bioelectrode were initially focused on investigation of the biosensor behavior in the solution of supporting electrolyte. Figure 4 presents cyclic voltammograms recorded in PBS obtained for the bare and TiO_2_/CMK-3/PDDA/Nafion electrodes (Fig. 4A, curves *a* and *b*, respectively) as well as for the TYR/TiO_2_/CMK-3/PDDA/Nafion biosensor (Fig. 4A, curve *c*). Two very broad redox peaks: anodic, stretching from ca. −110 to 150 mV, and cathodic distending from ca. −120 to 260 mV can be revealed on the current–potential curve registered for the biosensor (Fig. 4, curve *c*). Since no corresponding peaks were observed for the bare and non-enzymatic TiO_2_/CMK-3/PDDA/Nafion electrodes, the manifested pair of peaks could be attributed to the direct electron transfer (DET) between the catalytic center of tyrosinase and the electrode surface. Calculated formal potential, ca. 25 mV vs. Ag/AgCl (at pH 6), fitted in with the value of formal potential, 30 mV vs. SCE (at pH 7), obtained for wired tyrosinase mechanically compressed with MWCNT [38]. Nonetheless, for tyrosinase entrapped in titania sol–gel matrix modified, among others, multi-walled carbon nanotubes, a formal potential of 250 mV vs. Ag/AgCl (at pH 6) was reported [34]. The occurrence of direct electron transfer between the electrode surface and the active site of enzyme can be also observed on the CV curves registered in PBS at different scan rates, from 10 to 120 mV s^−1^ (Fig. 4B). In Fig. 4C, it is seen that the anodic (*I*_p,a_) as well as the cathodic (*I*_p,c_) peak currents were proportional to the scan rate suggesting a surface-controlled process. Direct electron transfer of TYR was reported to appear only for few biosensors: for that based on the enzyme covalently bound to glassy carbon electrode via Woodward’s reagent K [39], glassy carbon electrode modified, among others, with carbon nanotubes [34, 40, 41] and mesoporous carbon [42].

**Fig. 5:**
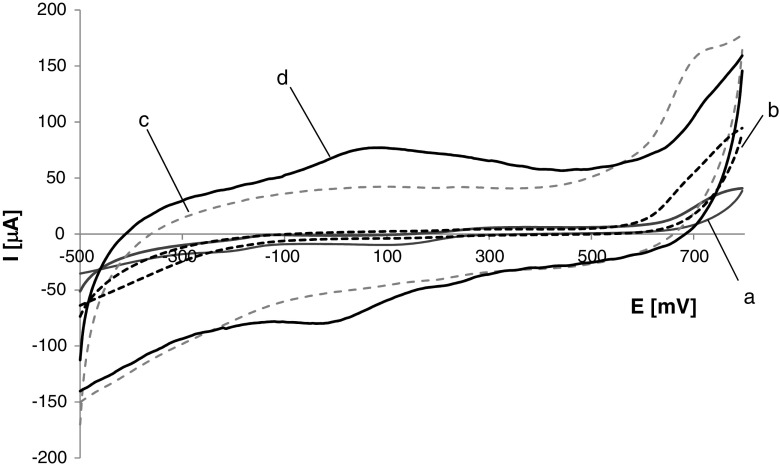
Cyclic voltammograms registered in 0.48 mM tyramine solution at TYR/TiO_2_ (**a**), TYR/TiO_2_/PDDA/Nafion (**b**), TYR/TiO_2_/CMK-3/Nafion (**c**), and TYR/TiO_2_/CMK-3/PDDA/Nafion biosensors (**d**); scan rate, 62.5 mV s^−1^

The electrochemical characterization of biosensors modified with different matrix composites was carried out in 0.48 mM tyramine solution. Figure 5 presents CV curves registered for the TYR/TiO_2_, TYR/TiO_2_/PDDA/Nafion, TYR/TiO_2_/CMK-3/Nafion, and TYR/TiO_2_/CMK-3/PDDA/Nafion biosensors (Fig. 5, curves *a* to *d*, respectively). It is found that biosensors based on matrices without mesoporous carbon exhibited narrow cyclic voltammograms (Fig. 5, a, b). The addition of CMK-3 into the matrix layer resulted in broadening of CV curves (Fig. 5, c, d), likely due to the increase of the effective surface area of the matrix composite. Nevertheless, a pair of broad redox peaks was noticed only for the TYR/TiO_2_/CMK-3/PDDA/Nafion biosensor (Fig. 5, d); no redox peaks were revealed for the other studied biosensors. The phenomenon confirmed that both PDDA and CMK-3 were necessary to develop the biosensor designed for tyramine quantification. PDDA, being positively charged, allowed entrapment of the large amount of negatively charged tyrosinase due to electrostatic attraction [43], while CMK-3 exhibiting a large surface area enhanced the amount of immobilized enzyme in its catalytically active form and ensured excellent conductivity of the biosensor matrix. The observed redox peaks could be attributed to redox processes of *o*-quinone, the product of enzymatic oxidation of tyramine (Scheme 1; see Supplementary Information). The effect of scan rates on cyclic voltammograms was investigated in the range from 10.0 to 120.0 mV s^−1^. The significant peaks’ width indicated slow redox processes, both oxidation and reduction. From Fig. S1A (see Supplementary Information), it is seen that the scan rate influenced the peaks’ potential position: along with the scan rate increase, the anodic peak, *E*_p, a_, moved to more positive potentials whereas the cathodic peak, *E*_p, c_, shifted to less negative potentials. However, the heights of the respective anodic and cathodic current peaks seem to be equal. These observations suggest quasi-reversible redox reactions. Both anodic and cathodic peak currents exhibited a linear relationship vs. scan rate (Fig. S1B; see Supplementary Information) indicating surface-controlled electrode processes, without diffusion limitation.

### Evaluation of analytical parameters of biosensor

**Fig. 6:**
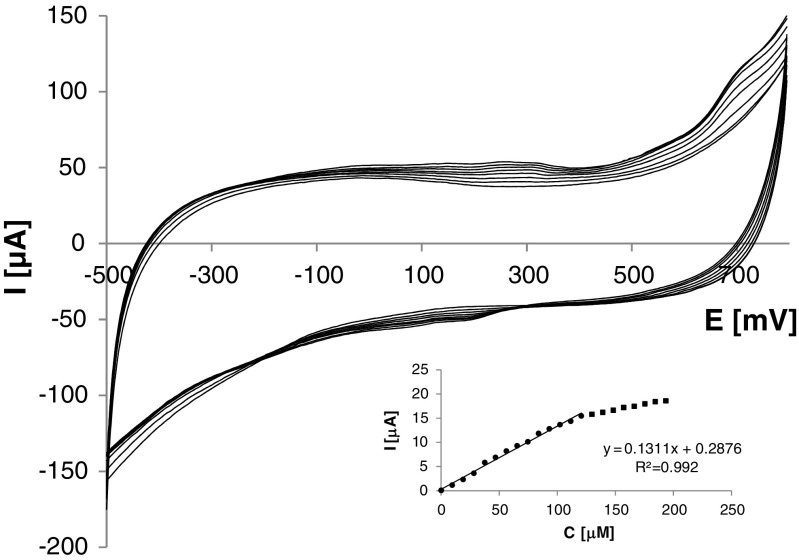
Exemplary cyclic voltammograms recorded at TYR/TiO_2_/CMK-3/PDDA/Nafion bioelectrode in background electrolyte containing tyramine at different concentrations; inset: linear range of biosensor response; scan rate, 62.5 mV s^−1^

The determination of tyramine was based on redox reactions of catalytically generated *o*-quinones taking place at an electrode surface (see Scheme S1; see Supplementary Information). Exemplary voltammetric responses of the developed biosensor recorded in analyte solutions of different concentrations are presented in Fig. 6. At the low tyramine concentration, the broad oxidation and reduction peaks are observed, over the potential range −150 to 400 mV. In solution with the slightly higher analyte content, the additional oxidation peak at ca. 650 mV was detected. That peak can be attributed to direct electrochemical oxidation of the analyte, whereas the anodic peak appearing at lower potential could be the result of tyramine oxidation related to enzymatic processes. According to this approach a very broad reduction peak could be assigned to reduction of both electrochemically and enzymatically generated *o*-quinones. The analogous interpretation of CV curves obtained for different phenols was reported for phenol oxidase-based bioelectric tongue [44]. For analytical purposes, enzymatically generated redox peaks were examined. Both peaks were easily noticeable and their heights were increasing along with concentration of analytes (see Fig. 6). It can be discovered that oxidation peak currents were higher than respective reduction ones, which indicated better sensitivity of determination based on biosensor oxidation response. Estimation of sensitivity obtained based on reduction and oxidation peaks gave mean values of 175 and 486 μA mM^−1^ cm^−2^, respectively. For that reason, quantification of tyramine was based on the biosensor response corresponding to the anodic peak current centered at ca. 200 mV. Selection of the comparatively low signal potential likely permits determination of analyte without influence of other electroactive compounds that can be found in natural samples and are usually electroactive at higher potentials [45]. Biosensor response shows linear dependency on tyramine concentration from 6 to 130 μM with mean sensitivity of 486 μA mM^−1^ cm^−2^ (136 μA mM^−1^). Limit of detection LOD = 1.5 μM was calculated based on the 3*s*/*a* formula, where *s* constitutes the standard deviation of signals recorded in 10 μM tyramine solution (*n* = 10) and *a* is biosensor sensitivity [46]. Repeatability and reproducibility were evaluated in 25 μM tyramine solution, and they can be considered satisfying. Biosensor repeatability, estimated as standard deviation (RSD%) of results (concentrations) obtained based on five independently received, for one bioelectrode, calibration sets, equaled 3.0 %. Reproducibility was obtained based on five calibration curves recorded individually for five independently prepared biosensors giving an RSD value of 3.1 %. For estimation of biosensor long-term stability, cyclic voltammetric response in 25 μM tyramine solution was investigated. There were no significant changes of recorded current signal after 7 days of biosensor storage in PBS at 4 °C, and after one additional week, the biosensor retained ca. 70 % of its initial response.

Enzymatic reaction at the developed biosensor suits well the Michaelis–Menten kinetic model. The apparent Michaelis–Menten constant, *K*_m_^app^, was calculated from the electrochemical version of the Lineweaver–Burk equation [45]:

$$ \frac{1}{I}=\frac{1}{I_{\max }}+\frac{K_{\mathrm{m}}^{\mathrm{app}}}{I_{\max }C} $$

where *I* is the steady-state current after addition of enzyme substrate (tyramine) and *C* constitutes tyramine concentration. The obtained value of *K*_m_^app^ = 66.0 μM is similar to the value reported for the biosensor with tyrosinase immobilized in phosphate-doped polypyrrole film, 62.65 μM [16] and lower than that obtained for the screen-printed electrode with enzyme entrapped in the carboxyl-functionalized single-walled carbon nanotube composite, 88.50 μM [17]. From the results, it can be concluded that the TYR/TiO_2_/CMK-3/PDDA/Nafion exhibited high biological affinity for tyramine.

The substrate specificity of the proposed biosensor was studied taking into account, as possible interferences, substances that are usually encountered in food products together with the analyte: other biogenic amines (glutathione, phenylalanine, and tryptophan), ascorbic acid, and inorganic ions (Mg^2+^, Ca^2+^, Na^+^, Cl^−^, NO_3_^−^). Furthermore, three phenolic compounds, substrates of tyrosinase, usually met in banana fruit [47]: gallic, vanilla, and caffeic acids, were taken into consideration. The measurements were performed in 50 μM tyramine solution. The collected results demonstrate that biogenic amines did not significantly influence the current signal up to 20-fold higher concentrations of glutathione and tryptophan, and 50-fold excess of phenylalanine. For inorganic ions, the tolerance ratio was 50. As expected, due to reducing properties of ascorbic acid, a noticeable increase of anodic and a decrease of cathodic peak currents were revealed at its concentration above 0.5 mM. A study on the interference effect of tyrosinase substrates showed that gallic acid did not change the analytical signal up to fivefold excess, while for vanilla and caffeic acids at a concentration of 50 μM, the current increase was revealed. Schematic presentation of the influence of interferences on analytical signal recorded in 50 μM tyramine solution is shown in Fig. S2 (see Supplementary Information). To evaluate the type of matrix effects and to determine accurate results employing integrated calibration [48] or generalized calibration strategy [49] could be suggested.

The performance of the proposed TYR/TiO_2_/CMK-3/PDDA/Nafion biosensor was compared with that of other biosensors designated for tyramine quantification. Taking into consideration the limit of detection, it can be stated (Table 1) that the developed biosensor exhibited lower LOD in comparison with biosensors based on tyramine oxidase [2, 15], monoamine oxidase [50], and pea seedling amine oxidase [4, 14] but higher than tyrosinase-based biosensors [16, 17] and the diamine oxidase-based bioelectrode [51]. Linear range is comparable to or narrower than ranges reported for other biosensors. Compared with other tyrosinase-based biosensors, the proposed biosensor exhibited high sensitivity toward the analyte, ca. 30 % higher than reported for biosensor with phosphate-doped polypyrrole matrix [16] but lower than that reported for a screen-printed electrode with enzyme entrapped in the carboxyl-functionalized single-walled carbon nanotube composite [17]. The limit of detection of the proposed biosensor, 1.5 μM, was higher than that described for other tyrosinase-based biosensors, 0.6 μM [16, 17]. Nonetheless, it is worth mentioning that from a toxicological point of view, only the consumption of a large amount of tyramine and other biogenic amines can be dangerous for human health. There is no legislation in the EU to limit the content of biogenic amines in food except from histamine in fish products (400 mg kg^−1^) [52]. Therefore, it seems that the developed biosensor exhibited a low enough LOD, 1.5 μM (0.21 mg L^−1^), to be useful for the determination of high tyramine concentration in food products. In support of this statement, the example of studies on in vitro cytotoxic effect of tyramine and other biogenic amines, at concentrations of 0.7 mM and higher, can be given [52].

**Table 1:** Comparison of analytical characteristics of electrochemical biosensors designated for tyramine determination

Electrode	Enzyme	Linear range [μM]	LOD [μM]	Reference
CPE/Nafion	Pea seedling amine oxidase	182–7290	45.2	[4]
SPCE/MnO_2_	Pea seedling amine oxidase	1–50	3.0	[14]
GE/Os mediator	Monoamine oxidase A/horseradish peroxidase	10–500	2.0	[47]
Pt	Diamine oxidase	1–50	0.5	[48]
Au/AgNPs/l-Cys	Tyramine oxidase	17–250	10.0	[2]
Pt /ERM	Tyramine oxidase	17–253	17.5	[15]
Pt/Ppy/PO_4_^3−^	Tyrosinase	4–80	0.6	[16]
SPCE/SWCNT-COOH	Tyrosinase	5–180	0.6	[17]
GE/TiO_2_/CMK-3/Nafion/PDDA	Tyrosinase	6–130	1.5	This work

*AgNPs* silver nanoparticles, *CPE* carbon paste electrode, *ERM* epoxy resin membrane, *GE* graphite electrode, *l* *-Cys* l-Cysteine, *Ppy* polypyrrole, *SPCE* screen-printed carbon electrode, *SWCNT-COOH* carboxyl-functionalized single-walled carbon nanotubes

On the other hand, it can be noticed that preparation of Tyr/SWCNT-COOH/SPE proposed by Apatrei et al. [17] requires use of vapor of glutaraldehyde, a very harmful reagent that can not only contribute to asthma symptoms, severe skin burns, and allergic skin reaction but is also very toxic to aquatic life with long-lasting effects. Construction of the proposed Tyr/TiO_2_/CMK-3/PDDA/Nafion biosensor also involves an unsafe reagent, Nafion, but it is used in solution and it is significantly less harmful than vapor of glutaraldehyde. Taking into account the roles of green chemistry and green analytical chemistry [53], the presented Tyr/TiO_2_/CMK-3/PDDA/Nafion biosensor should be considered as a greener device.

### Verification of biosensor functioning. Natural sample analyses

The developed biosensor was examined for its applicability in complex natural matrices, specifically in natural food products as Camembert cheese, sauerkraut, and banana. The volume of 0.5 mL of Camembert cheese and sauerkraut extracts or 1 mL of banana extract (see section “Sample preparation”) was taken for measurements. The analyses were performed using the standard addition method, based on six standard additions; each sample was analyzed three times. From Table 2, it is seen that the precision of the method can be regarded as satisfactory for natural samples, because the RSD value did not exceed 10 %. It was found that Camembert cheese contained 143.8 mg kg^−1^ whereas exemplary for Pecorino Romano cheese value of 116.7 mg kg^−1^ was reported [54]. The tyramine content in the sauerkraut sample was obtained to be 217.2 mg kg^−1^ compared with the value of 264.5 mg kg^−1^ detected by Apatrei [16]. The amount of tyramine in the fresh banana sample was calculated to be 121.2 mg kg^−1^.

**Table 2:** Quantification of tyramine in natural product samples by the proposed biosensor

Food sample	Tyramine [μM]	Average [μM]	RSD [%]
Camembert cheese	28.3	30.0	7.6
	28.4		
	33.2		
Sauerkraut	51.3	45.2	9.5
	42.4		
	42.0		
Fresh banana	26.7	25.3	4.7
	23.8		
	25.3		
Ripe banana	41.9	41.1	7.9
	36.8		
	44.6		

It is worth emphasizing that the tyramine content in food products varies and depends on such processes as ripening, fermentation, and degradation. In order to verify the usefulness of our biosensor in monitoring of ripening, part of the banana sample was left in the laboratory at room temperature for 4 days and after that time analyzed again. According to expectation, the tyramine content increased to 197.4 mg kg^−1^ proving that the proposed bioelectrode can be useful in screening of natural processes occurring in food.

To evaluate the efficiency of the proposed method, recovery studies were carried out. Examined food samples were spiked with 24.3 μM tyramine. From Table 3, it is seen that the proposed approach gave reliable results, as values 92.2, 93.0, and 102.1 % of recovery for Camembert cheese, sauerkraut, and banana samples, respectively, were obtained, with a mean recovery value of 95.8 %.

**Table 3:** Analytical recovery of added tyramine in the food samples

Real sample	Tyramine determined [μM]	Tyramine spiked [μM]	Tyramine detected [μM]	Recovery [%]	RSD [%]
Camembert cheese	30.0	24.3	52.4	92.2	7.3
Sauerkraut	45.2	24.3	67.8	93.0	4.5
Banana	25.3	24.3	50.1	102.1	8.2

## Conclusions

A voltammetric biosensor based on tyrosinase immobilized in the TiO_2_/CMK-3/PDDA/Nafion composite for the determination of tyramine in food products was proposed. The performed experiments proved that the mesoporous carbon material was an essential component of the matrix composite influencing the electrochemical biosensor response. The developed matrix composite ensured an adequate medium for entrapment of a large amount of tyrosinase in its catalytically active form that could be concluded from the phenomenon of direct electron transfer between the catalytic center of the enzyme and the electrode surface. The resulting biosensor presents good analytical characteristics in the quantification of tyramine, especially concerning high sensitivity and low value of LOD.

The present biosensor can operate as an amperometric device after optimization of the working electrode potential, both in batch and flow (e.g., FIA) modes. The optimal speed of steering should be found for batch conditions whereas appropriate volumes and flow rates of the sample and standard additions should be optimized for the FIA system.

It was demonstrated that without a complicated sample pretreatment and sophisticated apparatus, the biosensor can constitute an alternative method not only to determine tyramine in food but also to monitor food quality. Moreover, the developed biosensor matrix has the capability to be involved in the construction of other bioelectrodes.

## Electronic supplementary material

Below is the link to the electronic supplementary material.

ESM 1 (PDF 567 kb)
