# Supplementary material for: Mesoporous carbon-containing voltammetric biosensor for determination of tyramine in food products
Source: Anal Bioanal Chem. 2016 May 21;408:5199–210. doi: 10.1007/s00216-016-9612-y (PMC4925687; doi:10.1007/s00216-016-9612-y)
Supplement: Supplementary file 1 — (PDF 567 kb) [file 216_2016_9612_MOESM1_ESM.pdf]

Analytical and Bioanalytical Chemistry

Electronic Supplementary Material

Mesoporous carbon-containing voltammetric biosensor for determination of tyramine in food products

Jolanta Kochana, Karolina Wapiennik, Paweł Knihnicki, Aleksandra Pollap, Paula Janus, Marcin Oszajca, Piotr Kuśtrowski

[1] Jagiellonian University, Faculty of Chemistry, Ingardena 3, Krakow, Poland

Corresponding author: Jolanta Kochana

Tel. +48 126632014; fax. +48 126632232

E-mail address: kochana@chemia.uj.edu.pl

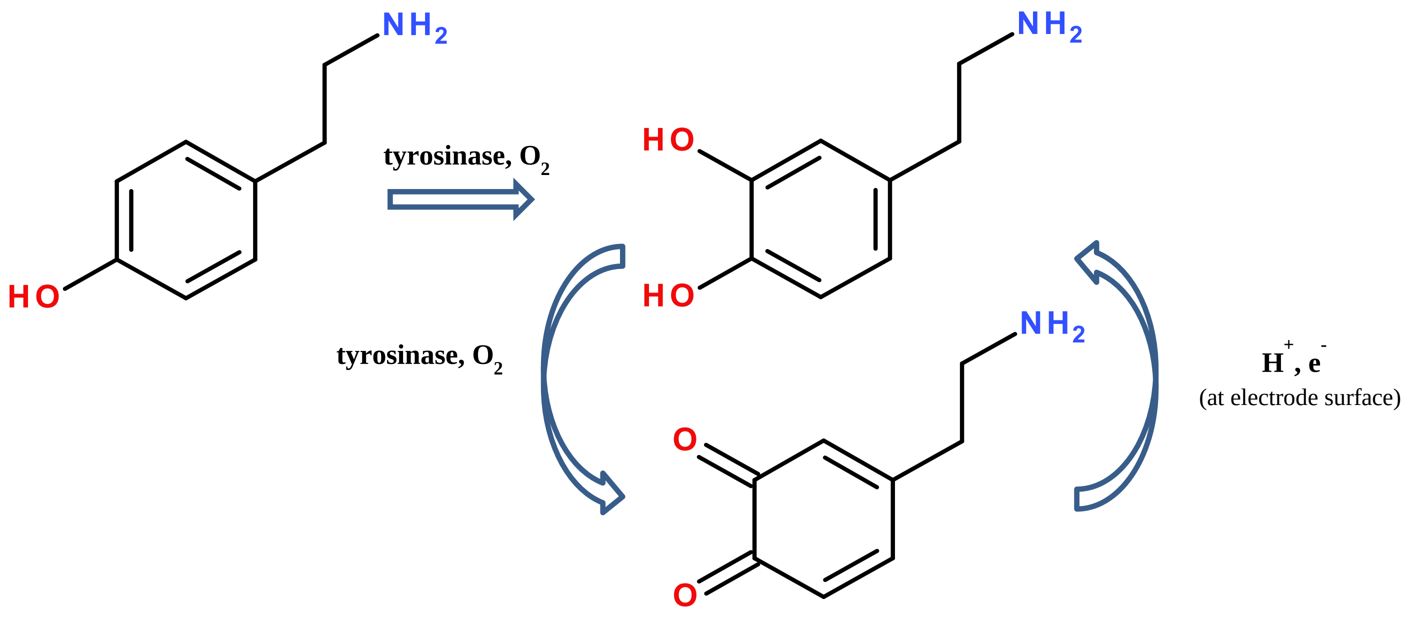

Scheme S1 Diagram of mechanism of enzymatic determination of tyramine at TYR/TiO2/CMK-3/PDDA/Nafion bioelectrode

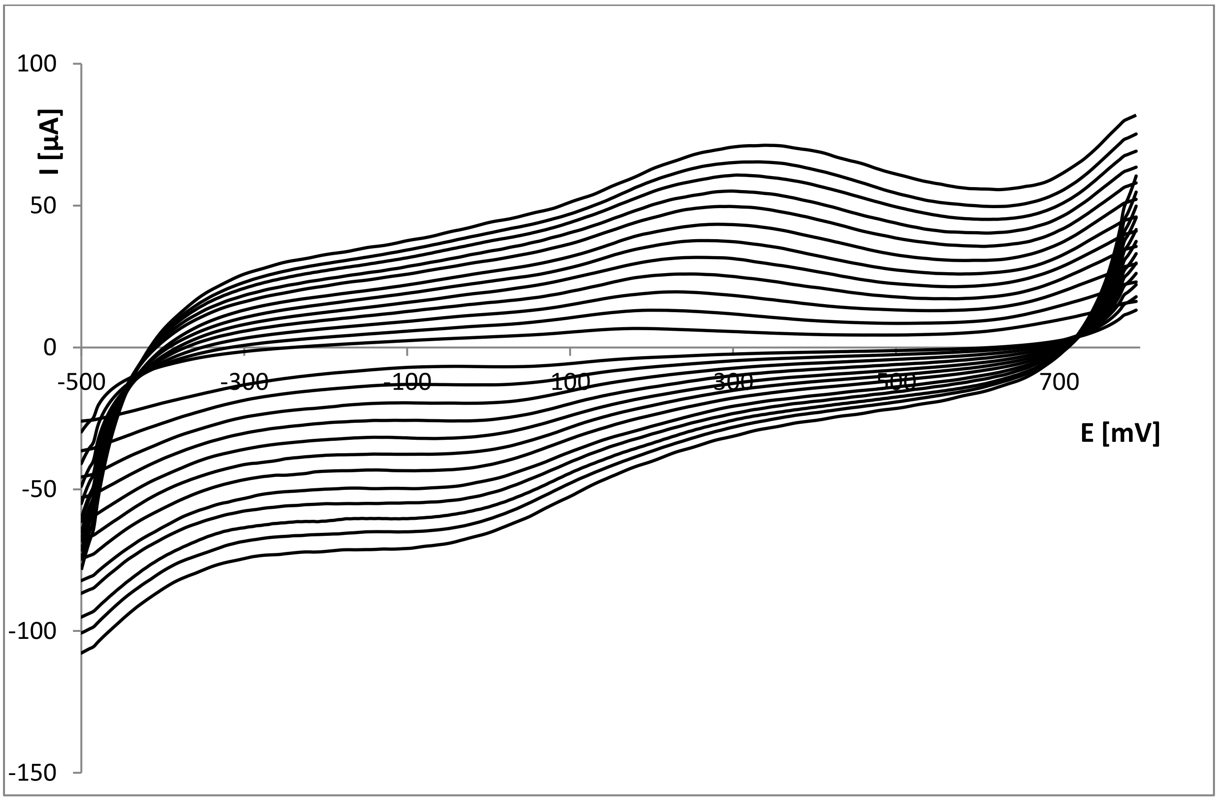

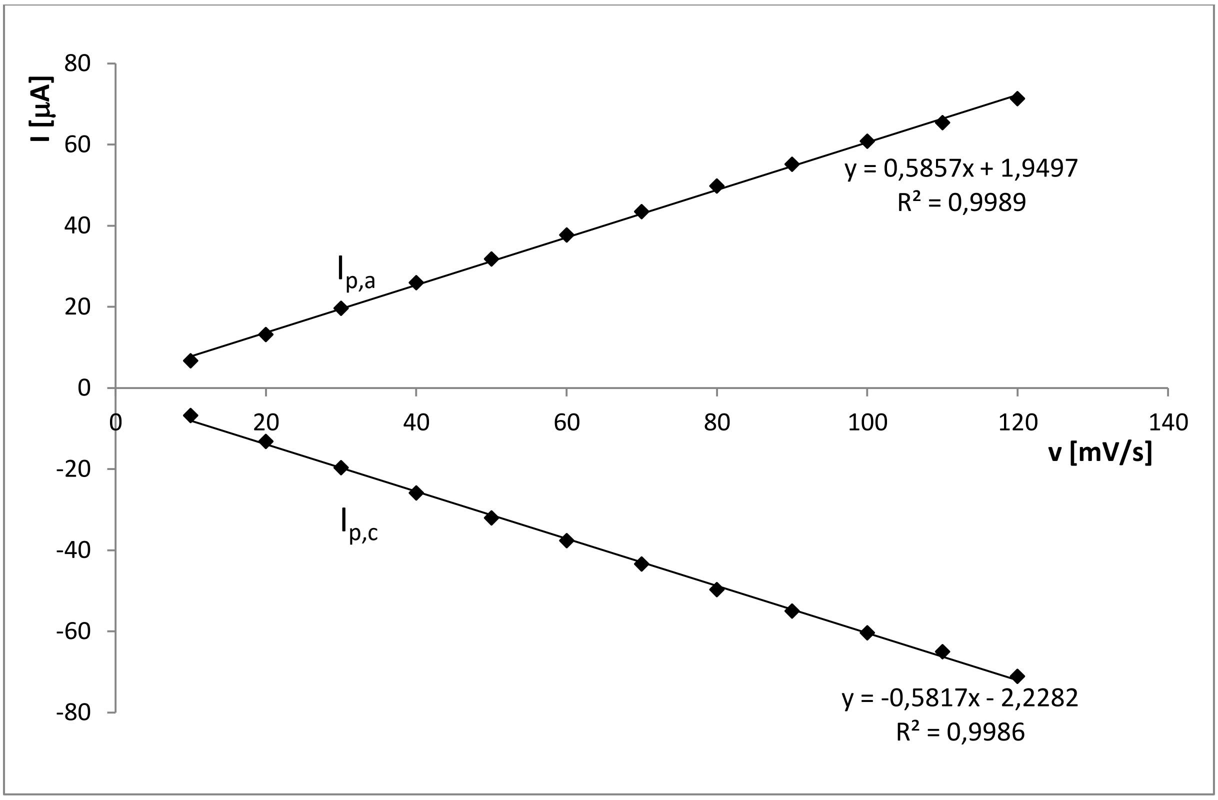

Fig. S1 Influence of scan rates on CVs recorded in 50 µM tyramine solution at TYR/TiO2/CMK-3/PDDA/Nafion biosensor (A); cathodic $I_{p,c}$ and anodic $I_{p,a}$ peak currents as a function of scan rate used (10-120 mV s^{-1}) (B)

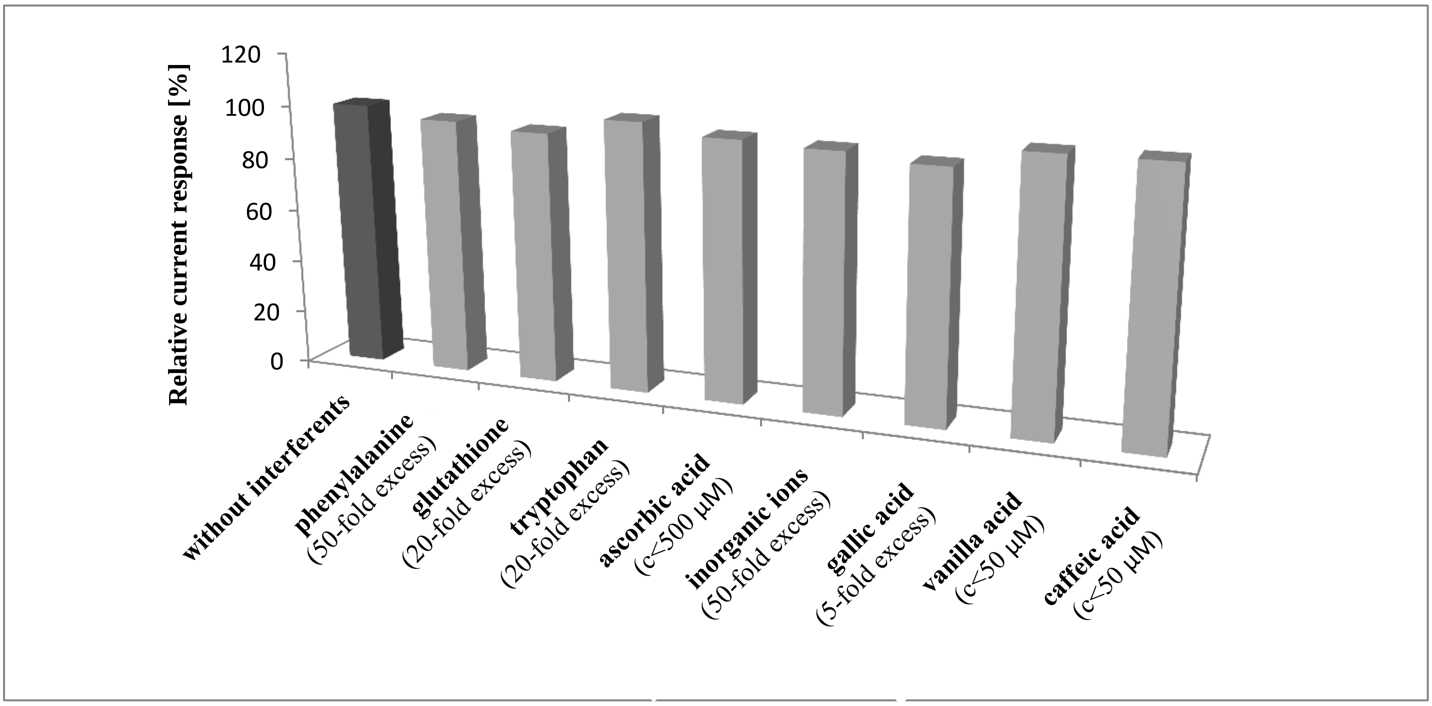

Fig. S2 Biosensor responses recorded in 50 μM tyramine solution in the presence of interferences
